# Quantifying subjective assessment of sleep quality, quality of life and depressed mood in children with enuresis

**DOI:** 10.1007/s00345-013-1193-1

**Published:** 2013-10-23

**Authors:** Oktay Üçer, Bilal Gümüş

**Affiliations:** 1 Uşak State Hospital, Urology Clinic, Uşak, Turkey; 2 Department of Urology, Faculty of Medicine, Celal Bayar University, Manisa, Turkey

**Keywords:** Enuresis, Depression, Sleep quality, Quality of life

## Abstract

**Aim:**

The aim of this study was to compare a group of children who has monosymptomatic nocturnal enuresis (MNE) with a healthy control group by assessing their depression scales, quality of life and sleep quality.

**Methods:**

Hundred and one children with MNE and 38 healthy controls are included in the study, aged between 8 and 16 years old. All participants were performed the Pediatric Quality of Life Inventory (PedsQL 4.0), Depression Scale for Children (CES-DC) and The Pittsburgh Sleep Quality Index (PSQI) tests. The two groups were compared for their demographic factors and for the results of the tests above.

**Results:**

There were no significant differences between the two groups according to age, gender and other demographic factors. Quality of life, depression and sleep quality scores implied worse health in the patient group. The PedsQL scores were assessed as 1,659.90 ± 296.01 in the patient group and 1,818.42 ± 227.92 in the control group (*p* = 0.001). The CES-DC scores were 11.74 ± 6.11 in the patient group and 7.00 ± 3.97 in the control group (*p* < 0.001). And the PSQI scores were 2.58 ± 2.48 in the patient group, 1.15 ± 1.10 in the control group (*p* < 0.001). Also in the patient group, there was a positive correlation between participants’ ages and the PedsQL (*p* = 0.010; *r* = 0.256), the CES-DC (*p* = 0.000; *r* = 0.382), the PSQI (*p* = 0.000; *r* = 0.403) scores. The success parameters at school were significantly worse in the patient group (*p* = 0.05).

**Conclusion:**

All our findings show us that the children with nocturnal enuresis were affected negatively because of their disease; especially when they grow up the scores get worse health, so we suggest that treatment must be started at suitable age according to guidelines.

## Introduction

According to the International Children Continence Society, nocturnal enuresis (NE) is defined as discrete incontinence episodes while an individual is asleep [1]. This terminology is applicable to children who are at least 5 years old. Enuresis in children without any other lower urinary tract symptoms (such as increased/decreased voiding frequency, daytime incontinence, urgency, hesitancy, straining, a weak stream, intermittency, holding maneuver, a feeling of incomplete emptying, post-voiding dribble and genital or lower urinary tract pain) and without a history of bladder dysfunction is defined as monosymptomatic nocturnal enuresis (MNE). Primary nocturnal enuresis (PNE) applies for children who have not achieved a previous dry period of at least 6 months [2].

There is no one universally successful treatment for NE. Behavioral therapy should be offered to all children with bed-wetting, but this therapy can be a little hard to be applied. Successful behavioral therapy requires a supportive parent, a motivated child, patience and an average of about 6 months of therapy [3]. Bed-wetting alarm therapy is another alternative for the cure of NE. As a medical therapy, desmopressin acetate offers an effective control of NE and is now available in a melt preparation that offers an improved safety profile. There is not any consensus about which therapy to choose in which patient yet. Some authors support the medical therapy; however, others suggest that only behavioral therapy is enough for the treatment [4].

Nocturnal enuresis has been accepted as a bio-behavioral problem common in early childhood that becomes more uncommon after the age of 7 years [5]. The overall prevalence of NE declines with increasing age, occurring in 7 % of children aged 10 years, 6 % aged 11 years and 0.5 % aged ≥18 years [6]. Most of these children grow out bed-wetting without taking any therapy. This is a result of the increasing number of children who spontaneously achieve nighttime bladder control. Current data suggest an annual healing rate of 15 % [7]. Therefore, it is difficult to decide to give medical therapy or not. Evaluation of depression mood, life and sleep quality in children with enuresis may help us to decide this issue. We aimed to assess the depression scale, life and sleep quality of these children, so we will offer the clinicians to apply medical therapy or not according to result of these tests.

## Materials and methods

### Subjects and study design

The study comprised of 101 consecutive children (62 boys and 39 girls) with MNE who had attended the urology clinic as outpatients and 38 healthy controls. Clinical diagnosis for the patient group was based on a careful history. Volunteer children in the control group were selected consecutively among healthy children of our hospital staff. The children in the control group were the same age group as the children in the patient group. Patients with any chronic disease that would affect their sleep quality (e.g., tonsil hypertrophy, obstructive sleep apnea symptoms) or patients with bladder dysfunction (daytime symptoms such as urge, frequent or infrequent voiding and daytime wetting) were excluded from the study. Patients who had received medication for enuresis before the study or patients with psychological disease such as autism, depression or anxiety were also excluded.

To exclude urologic or neurologic abnormalities, we took a detailed case history, and a physical examination was carried out. In addition, routine chemical analysis of blood, urine culture and ultrasound measurements of residual urine volume were conducted. No patient was excluded due to findings of ultrasound, chemical analysis and urine culture.

### Instruments

Depression, quality of life and sleep quality of the children in this study were assessed by using Children’s Depression Inventory (CDI), The Pittsburgh Sleep Quality Index (PSQI) and Pediatric Quality of Life Inventory (PedsQL 4.0).

Subjective assessment of sleep was determined with the PSQI questionnaire. This scale is used to quantify the quality of sleep over the past month. It was devised to provide a standardized measurement of sleep quality. The scale is straightforward and consists of 19 self-assessed items grouped into seven components weighted 0–3. The overall score ranges from 0 to 21, with lower scores indicating better quality of sleep. An overall score >5 on the PSQI indicates serious problems relating to at least two components, or moderate difficulties relating to more than three components [8]. Turkish version of the PSQI was validated by Agargün et al. [9].

The CDI is a 27-item self-report inventory of childhood depression that taps a variety of depressive symptoms. Items assess negative mood, interpersonal difficulties, negative self-esteem, ineffectiveness and anhedonia in children ages 7–17 years. Each item offers respondents three alternatives scored 0, 1 or 2 and accordingly raw scores range from 0 to 54. Several studies recommended 13 as a cutoff score for clinical populations and 19 as the threshold for community samples in the USA [10]. Turkish version of the CDI was validated by Öy, and the cutoff score of the CDI was recommended as 19 in Turkey [11].

The PedsQL™ 4.0 Generic Core Scales consisted of 23 items divided into four subscales including physical, emotional, social and school functions with 8, 5, 5 and 5 items, respectively. Likert response scale with five categories was used, ranging from never a problem (0) to almost always a problem [4]. All the subscales were transformed to a 0–100 score so that higher scores represented better quality of life. Turkish version of the PedsQL was validated by Sonmez et al. [12].

### Statistical methods

Student’s *t* and Chi-square tests were used for comparison between data of the patient and control groups. Pearson’s or Spearman’s correlation analyses were used to evaluate relationships between various variables. Statistical analyses were carried out using the SPSS 13.0 (SPSS Inc). *P* value <0.05 was considered significant.

## Results

Sociodemographic attributes of the children in the patient and control groups are summarized in Table 1. Although there were no significant differences between the two groups according to gender and housing type, the school success of the children in the enuretic group was significantly poorer than the control group. Besides, there were significant differences between the two groups according to NE history of all the family members (mother, father, sibling and second-degree relatives) (Table 1).

**Table 1:** Sociodemographic characteristics of the children and their families in the study groups

	Enuretic group (*n* = 101) (%)	Control group (*n* = 38) (%)	*p* value
Gender			
Male	62 (61.4)	24 (63.2)	0.84
Female	39 (38.6)	14 (36.8)	
School success			
Very good	48 (47.5)	24 (63.2)	0.05*
Good	27 (26.7)	12 (31.6)	
Moderate	20 (19.8)	2 (5.3)	
Poor	6 (5.9)	0 (0)	
PNE history of mother			
Yes	22 (21.8)	0 (0)	0.002*
No	79 (78.2)	38 (100)	
PNE history of father			
Yes	26 (25.7)	2 (5.3)	0.007*
No	75 (74.3)	36 (94.7)	
PNE history of sibling			
Yes	14 (13.9)	0 (0)	0.02*
No	85 (84.2)	34 (89.5)	
PNE history of second-degree relatives			
Yes	46 (45.5)	6 (15.8)	0.001*
No	55 (54.5)	32 (84.2)	
Are the parents married?			
No	6 (5.9)	4 (10.5)	0.46
Yes	95 (94.1)	34 (89.5)	
Housing type			
Urban	71 (70.3)	30 (78.9)	0.56
Rural	30 (29.7)	8 (21.1)	

	Mean ± SD (min–max)	Mean ± SD (min–max)	*p* value
Age (years)	10.94 ± 2.07 (8–16)	11.05 ± 1.93 (8–14)	0.77
Mothers age (years)	34.78 ± 5.30 (23–50)	35.26 ± 4.02 (30–45)	0.61
Fathers age (years)	38.39 ± 6.19 (26–63)	38.94 ± 3.88 (32–49)	0.61
Number of siblings	1.47 ± 0.83 (0–4)	1.42 ± 0.82 (0–3)	0.73
Number of wet nights (nights/weeks)	4.84 ± 2.18 (2–7)	–	

*PNE* primary nocturnal enuresis

*p < 0.05 was considered statistically significant

Though the mean age of two group were statistically similar (*p* > 0.05), quality of life, depressed mood and sleep quality were significantly worse in the enuretic group. The PedsQL scores were assessed as 1,659.90 ± 296.01 in the enuretic group and 1,818.42 ± 227.92 in the control group (*p* = 0.001). Although the physical and school scores of the PedsQL in the enuretic group were significantly lower than the scores of the control group, emotional and social scores were statistically similar in the groups (Table 2).

**Table 2:** Comparison of the scales of the children in the groups

	Enuretic group (*n* = 101)	Control group (*n* = 38)	*p* value
	Mean ± SD (min–max)	Mean ± SD (min–max)	
CDI	11.74 ± 6.11 (1–30)	7.00 ± 3.97 (1–16)	<0.001*
PSQI	2.58 ± 2.48 (0–10)	1.15 ± 1.10 (0–4)	<0.001*
PedsQL-total	1,659.90 ± 296.01(1,050–2.250)	1,818.42 ± 227.92(1.375–2,200)	0.001*
PedsQL-physical functioning	566,58 ± 125.26 (300–800)	622.36 ± 97.91 (450–800)	0.007*
PedsQL-emotional functioning	366.83 ± 93.48 (75–500)	380.26 ± 78.23 (250–475)	0.39
PedsQL-social functioning	408.66 ± 77.89 (200–500)	422.36 ± 80.91 (250–500)	0.37
PedsQL-school functioning	305.44 ± 97.24 (0–500)	392.10 ± 41.14 (300–475)	<0.001*
Time to fall asleep (min)	14.22 ± 10.15 (3–45)	11.73 ± 9.18 (2–30)	0.18
Sleeping time (h)	8.87 ± 1.02 (6–11)	9.13 ± 0.61 (8–10.5)	0.07

*CDI* Children’s Depression Inventory, *PSQI* Pittsburgh Sleep Quality Index, *PedsQL* Pediatric Quality of Life Inventory

*p < 0.05 was considered statistically significant

Both CDI and PSQI scores of the enuretic group were found significantly higher than the scores of the control group. The mean of the CDI scores was 11.74 ± 6.11 in the enuretic group and 7.00 ± 3.97 in the control group (*p* < 0.001), and the mean of the PSQI scores was 2.58 ± 2.48 in the enuretic group and 1.15 ± 1.10 in the control group (*p* < 0.001). There were no significant differences between the groups according to sleeping time and time to fall asleep.

In the enuretic group, there was a positive significant correlation between the children’s ages and the PedsQL (*p* = 0.010; *r* = 0.256), CDI (*p* = 0.000; *r* = 0.382) and PSQI (*p* = 0.000; *r* = 0.403) scores.

## Discussion

Monosymptomatic nocturnal enuresis is a common problem that can be troubling for children and their families. Also, bed-wetting has many different consequences on the development of the behaviors and the psycho-social state of the effected individuals [13]. The issue of emotional or behavioral disturbance in enuretics has been investigated in many controlled studies [14]. However, depression and quality of life of children with MNE have not been assessed in these studies. The therapy of enuresis may be overtreatment for the children with MNE because most of these children grow out bed-wetting without any therapy. It is important to determine effect on psychological state, sleep quality and quality of life of bed-wetting in enuretics.

Quality of life of children with MNE has only been assessed by Ertan et al. [15] in the literature. They used KINDL QoL to evaluate quality of life of the children and found that there was no significantly difference between the enuretic (*n* = 44) and control (*n* = 27) groups. In contrast, we found that quality of life of the children with enuresis (*n* = 101) was significantly worse than the children in control group (*n* = 38). Both the total and subgroups (physical and school) scores of PedsQL in the enuretic group were statistically lower than the scores in the control group. The reason for this may be the difference between numbers of the children in the two studies. The questionnaires used to assess quality of life were also different in these studies.

The association between MNE and psychological disorders has been evaluated in many studies [16, 17]. These studies are generally population based, and depressed mood in children with enuresis has not been essentially assessed. We investigated depressed mood in the children with MNE as compared to control subjects and found that the children with MNE were unhappier than the healthy children. While several studies recommended 13 as a cutoff score according to CDI, the authors in the USA and our country recommended 19. In the present study, the mean CDI score in the enuretic group was found 11.74, which is below the cutoff scores. As a consequence, the children with MNE in our study had not depression, but they were unhappy because of their disease.

Sleep quality of children with MNE was evaluated with PSQI and found worse than control subjects by Gozmen et al. [18]. We found that the sleep quality of the enuretics was worse as well. Sleep measures of children with MNE were subjectively derived from 3 to 5 nights of actigraphy and daily logs by Cohen-Zrubavel et al. [19]. They obtained similar results. In the present study, although sleep quality was worse in the enuretic group, sleep time and time to fall asleep were similar in both groups. All results show that children with MNE sleep worse. The reason for this may be hygiene problems such as waking up to take a bath, changing clothes or changing bed lining.

We found that though the CDI and PSQI scores in the enuresis group significantly increased with rising age, the PedsQL scores significantly decreased. These data show that younger children are less affected by bed-wetting than older children. Further studies may determine at what age children with MNE are getting to be suffered from bed-wetting. We think this issue may be important to decide at which age active treatment should be started. Younger children with MNE may not take any therapy if their parents are not annoyed in this circumstance. Therefore, these children will not take overtreatment because of growing out bed-wetting without any therapy.

This study had some limitations. A major limitation is that there was no adjustment for potential confounders. Poor quality of life of the patients may be caused by some factors such as age, gender, socioeconomic status or school status. Although there was no statistically difference according to gender, age and housing type between two groups, school success of the enuretics was poorer. Therefore, poor quality of life may be especially caused by school status.

## Conclusion

Our study indicates that the children with MNE are unhappy and have impaired quality of life and sleep quality because of bed-wetting, and this uncomfortable situation is progressing with rising age. We suggest that children with MNE who are 8 or older should be taken therapy because the age range in our study was from 8 to 16. Further studies may found an appropriate cutoff age to begging therapy in enuresis.
